# A modified vision transformer framework for image-based land cover segmentation in rural architectural design and planning

**DOI:** 10.1038/s41598-025-19234-w

**Published:** 2025-09-23

**Authors:** Sobia Wassan, Anas Bilal, Abdulkareem Alzahrani, Khalid Almohammadi, Malek Alrashidi, Seyed Jalaleddin Mousavirad

**Affiliations:** 1 School of Equipment Engineering, Jiangsu Urban and Rural Construction Vocational College, Changzhou, 213000 China; 2 College of Information Science and Technology, Hainan Normal University, Haikou, 571158 China; 3 Computer Science Department, Faculty of Computing and Information, Al-Baha University, 65779 Al-Baha, Saudi Arabia; 4 Computer Science Department, Applied College, University of Tabuk, Tabuk, 71491, Saudi Arabia; 5 Department of Computer and Electrical Engineering, Mid Sweden University, Sundsvall, Sweden

**Keywords:** Image segmentation and processing, Modified vision transformer, Modified firefly algorithm, Monte carlo simulation, EuroSAT dataset, Engineering, Mathematics and computing

## Abstract

Sustainable development, cultural preservation, and rural quality of life depend on rural architectural design and planning. It balances modern infrastructure needs with rural ecological and social characteristics to ensure sustainable growth. This research presents a deep learning framework based on a modified and robust vision transformer optimized with a modified firefly algorithm to perform image-based land cover segmentation in rural architectural design and planning. The proposed model formulates a high-dimensional feature space from fixed-size patches, then performs positional encodings and transformer encoder layers to maintain spatial information within these patches. Then, it embeds the self-attention mechanism of these layers to capture the interdependence of global spatial regions, which is crucial for bifurcating the region of interest. The optimization of the features and selection of hyper-parameters are performed with a modified firefly algorithm. The framework is validated using the EuroSAT benchmark dataset, derived from Sentinel-2 satellite imagery, consisting of 27,000 geo-referenced samples across 10 balanced land cover classes such as annual crop, forest, herbaceous vegetation, highway, and residential areas. Each image has a spatial resolution of 64 × 64 pixels in RGB format, ensuring consistent representation of rural and urban features. The quality of the segmented regions achieved an overall accuracy of 99.5% with a kappa coefficient of 0.81, while the mean fitness value was 4.9930 × 10^–8^ on the publicly available rural dataset. The results are compared with state-of-the-art, pre-trained existing models like VGG-19, ResNet-50, DarkNet-19, Inception-V3, and other reported techniques like Gaussian Naïve Bayes, geosystem approach, deep CNN and fuzzy DL hybrid with chaotic PSO that have the classification accuracy of 89.96%, 91.52%, 86% and 93.3%, respectively. The stability of the proposed model is investigated through Monte Carlo simulation based on 200 independent runs and their statistical investigations. The proposed predicted system is quite helpful for rural areas’ transformation into urbanization by mitigating climate change, natural resources and geographical characteristics.

## Introduction

Rural architectural design and planning (RADP) is crucial for developing sustainable, functional, and culturally attuned rural environments. As of 2023, rural regions comprise 44% of the global population and occupy 80% of the Earth’s landmass, according to the United Nations^1^. Planning in these regions must account for agricultural fields, forests, wetlands, and residential zones. To achieve resilient designs, rural planning must incorporate quantitative data such as soil health, water availability, and climate patterns, as more than 10% of rural areas experience land degradation due to unsustainable practices. Geographic Information Systems (GIS) and remote sensing (RS) are employed to delineate land utilization, improve resource distribution, and assess the ecological effects on human settlements, thereby improving rural spatial organization^2^.

Rural architecture must harmonize traditional construction techniques with modern technologies to mitigate environmental impact; in this regard, the International Renewable Energy Agency states that utilizing locally sourced bamboo, clay, or stone can reduce construction expenses and carbon emissions by 30%^3^. Passive cooling systems and renewable energy integration facilitate sustainable development by decreasing energy consumption in rural homes by 40%^4^. Rural roads constitute 60% of global road networks, so their planning enhances infrastructure to link agricultural products to markets^5^. Rural architectural design may improve quality of life, preserve ecosystems, and foster climate resilience through the utilization of accurate geospatial data and innovative construction techniques.

The rural design prioritizes simplicity, sustainability, and functionality, embodying local customs, environmental harmony, and agricultural lifestyles. Vernacular designs utilizing locally sourced materials such as wood, stone, adobe, and bamboo render houses environmentally sustainable and well suited^6^. Open floor layouts, interaction with nature, passive cooling, rainwater harvesting, and community-oriented spaces such as courtyards and barns are defining features of rural design. Rural areas have low population density, are predominantly employed for agricultural or conservation purposes, possess minimal infrastructure, adhere to certain zoning restrictions, and feature extensive open spaces with limited urban impact^7^. Natural integration, self-sufficiency, and cultural preservation differentiate rural architecture from urban architecture, which prioritizes modernization, high-density constructions, industrial materials, and technological advancements. Rural architecture is characterized by openness, greenery, and a focus on community, rendering it vital for sustainable and heritage-conscious development^8^.

In this regard, image-based land cover segmentation provides quantitative insights to address global challenges such as deforestation, urban expansion, and climate change by which the policymakers can monitor environmental alterations, strategize sustainable agriculture, and identify erosion and flooding hotspots through the analysis of segmented maps^9^. Precisely delineating agricultural fields facilitates irrigation management while recognizing natural barren areas that are important for rural development. Moreover, these maps provide precise actions for climate resilience and biodiversity conservation, necessitating a high degree of accuracy in the domain of agricultural and rural sustainable development. These segmented maps classify and distinguish land-cover types such as forests, water bodies, and urban areas in satellite or aerial imagery^10^ Over traditional segmentation techniques, the methods of machine learning (ML) have shown dominancy in terms of applicability, accuracy, effectiveness and stability to handle large-scale data, complex datasets and distortion in RS information. In this regard, supervised learning is used for regression and classification problems that have labelled datasets.

In contrast, unsupervised learning is suitable for dimension reduction and clustering where the images have correlated attributes, while reinforcement learning best suits the problems of control and classification^11,12^. Considering the difficulties associated with RADP, the RS based rural area dataset well known in the literature as EuroSAT^13^ is taken. The main attributes include spatial resolution, number of images in each Class, number of classes and inter-class interference^14^. To address these challenges, The modern techniques of deep learning (DL) such as convolutional neural networks (CNN), pre-trained models, U-shaped Networks (U-Net), deep CNNs for semantic image segmentation (DeepLab), and mask recurrent CNN (R-CNN) precisely categorizes the land cover from high-resolution geospatial images to achieve high overall accuracy and Intersection over Union (IoU) for multiclassification^15,16^. Contemporary segmentation models can achieve a mean Intersection over Union (mIoU) of over 85% on EuroSAT and DeepGlobe Land Cover^17^, rendering them indispensable for extensive land use planning and resource management.

Rural architecture analysis uses classical picture segmentation algorithms, including thresholding, region-based segmentation, and edge detection, as well as deep learning-based methods like CNNs, U-Net, Mask R-CNN, and Vision Transformers (ViTs). Canny and Sobel’s filters help with basic feature extraction, but varied textures and fluctuations in rural settings make thresholding and edge detection difficult. Region-based approaches segment cropland, vegetation, and rural structures but are noise-sensitive^18^. Rural land-use classification commonly uses machine learning methods like K-means clustering and Random Forest segmentation. Recent deep learning models like U-Net and Mask R-CNN use CNNs for high-precision segmentation, making them excellent for spotting rural structures, roads, and agricultural fields. ViTs and Swin transformers are also used for high-resolution rural picture segmentation, improving boundary recognition, vegetation mapping, and structural classification^19^. These advances improve rural land planning, conservation, and historical preservation.

However, there is always a need for a reliable, efficient and precise DL model to perform better segmentation than that of existing techniques. The list of the abbreviations used in the study is presented in Table 1.

**Table 1:** List of the abbreviations “Abbre”.

Description	Abbre	Description	Abbre
Rural architectural design and planning	RADP	Vision transformer	ViT
Geographic information systems	GIS	Squeeze and excitation	SE
Convolutional neural network	CNN	Intersection over union	IoU
Deep learning	DL	Random forest	RF
Machine learning	ML	Remote sensing	RS
Rural architectural design and planning	RADP	mean intersection over union	mIoU
Land cover segmentation	LCS	Multi headed self-attention	MFSA

Moreover, the list of the symbols is also tabulated in Table 2 for clarity.

**Table 2:** List of the symbols.

Description	Symbols	Description	Symbols
Annual crop	AC	Forest	F
Highway	H	Industrial	I
Pasture	P	Residential	Re
Inertia factor	$\varpi$	Weight matric	${w}_{1},{w}_{2}$
Mean	$\mu$	kurtosis learning rate	$\widehat{\kappa }$
Random parameter	$\alpha$	River	$\xi$ R
Standard deviation	$\sigma$	kappa coefficient	$\Xi$
Excitation channel	${f}_{c}$	Sea lake	SL
Predefined frequency	${P}_{f}$	Positional encoding	${\mathcal{T}}_{0}$
Herbaceous vegetation	HV	Permanent crops	PC
Absorption coefficient	$\gamma$	GeLU activation	$\phi$
Light intensity	$\delta$	squeezed channel value	$s$
Global average pooling	${g}_{p}$	Fully connected operation	${f}_{1}$
Cls-indexing layer	${\mathcal{T}}_{CLS}$	Fully connected layer	${\mathcal{T}}_{FC}$
Highest brightness	${\lambda }_{o}$	Geometric factor	$\psi$
Width of the feature map	$W$	Height of feature map	$H$
Recalibrated activation	${\mathcal{T}}_{a,b,p}$	Distance b/w firefly	*d*

The main research contributions are listed below:

- A robust modified vision transformer (ViT-SE) is formulated that has high-dimensional feature space from fixed-size patches, then performs positional encodings through transformer encoder layers to maintain spatial information within these patches. It embeds the self-attention mechanism of these layers to capture the interdependence of global spatial regions, which is crucial for detecting complex and overlapping segments of the other classes in the classification of data that is required for a sustainable RADP.
- A modified firefly algorithm (MFA) is designed by introducing inertia factor “$\psi$” and diversity in the standard firefly algorithm to optimize Vit-SE architecture hyperparameters to have reduced learnable parameters.
- The results of the proposed framework are compared with state-of-the-art pre-trained models like VGG-19, ResNet-50, DarkNet-19, Inception-V3, and other reported contributions based on Gaussian Naïve Bayes^20^, geosystem approach^21^, deep CNN^22^ and fuzzy DL hybrid with chaotic PSO^23^.
- The stability of the proposed model is ensured using Monte Carlo simulations of 200 independent runs based on their fitness value and, consequently, global values of overall accuracy, F1-score, recall and precision.
- Moreover, the reliability of the model is guaranteed based on the analysis of different statistical measures like minimum, maximum, mean, standard deviation, and kappa factor. The computational complexity is also computed to see the significance of the proposed approach.

The research questions (RQ) established for the research carried out are given below:

### RQ1

How does the proposed model enhance high-dimensional feature extraction and spatial information retention for sustainable RADP?

### RQ2

How does the integration of the self-attention mechanism in ViT-SE improve the detection of complex and overlapped segments in classification tasks?

### RQ3

What impact do the inertia factor (ψ) and diversity enhancement in the MFA have on optimising ViT-SE hyperparameters and reducing learnable parameters?

### RQ4

How does the ViT-SE framework compare with state-of-the-art deep learning models (e.g., VGG-19, ResNet-50, DarkNet-19, Inception-V3) and other reported methods in terms of classification accuracy and robustness?

### RQ5

How stable is the proposed ViT-SE + MFA framework across multiple independent trials, as evaluated through Monte Carlo simulations and key performance metrics?

### RQ6

How does the computational complexity of the ViT-SE + MFA framework compare with traditional deep learning models, and what significance does it hold for real-world applications in rural architecture segmentation and classification?

The rest of the article is organised in a way that a detailed literature review, along with the gap analysis and reported work on the same dataset, is given in Section II. The proposed ViT-SE model, MFA, pseudocode, details of the dataset, and performance measures used in the article are presented in the Materials and Methods section, along with the nature of the dataset used in the study. The hyperparameters optimised through MFA, software and hardware used for the simulation, and the results of the proposed framework are provided in Section IV. In contrast, the conclusion, limitations of the framework and directions of the future work are drawn in the last section.

## Related work and the state of the art

Recent advancements in multiple fields, ranging from remote sensing (RS), machine learning (ML), and deep learning (DL), have brought environmental monitoring, classification, and segmentation to cutting-edge achievements. In the broader field of remote sensing, image-based land cover segmentation (LCS) and environmental monitoring, several recent studies have contributed to progress in RS and computer vision owing to ML and DL techniques. For instance, layout optimization approaches have been applied for agriculture and small-scale agrarian industries^24^, while the smart hydroponics farming systems have been developed using novel genetic algorithms^25^. Advanced DL-based vehicle detection and classification in remote sensing imagery has been enhanced by chaotic equilibrium optimization^26^. In urban and land cover change detection, STCD-EffV2T Unet has demonstrated improved performance using Sentinel-2 satellite images^27^. Similarly, building damage detection has benefited from UNet-GCViT, incorporating global context vision transformer blocks^28^, and EMYNet-BDD, which combines EfficientViTB with YOLOv8 in an encoder–decoder architecture^29^.

Recent advancements in image processing, remote sensing, and intelligent vision systems have significantly transformed applications across marine engineering, aerospace, robotics, and environmental monitoring. For instance, studies have proposed novel frameworks for crack detection in broken ice, bathymetric LiDAR data acquisition, and the lunar crater reconstruction, enhancing the precision of geospatial analysis^30–32^. In security and safety contexts, anti-overlapping detection in X-ray imaging, low-light enhancement via dehazing models, and unsupervised change detection approaches have been introduced^33–35^. Remote sensing research has further contributed through pixel dichotomy models for vegetation cover estimation, Sentinel-2 imagery-based vegetation mapping, and multi-modal image fusion techniques^36–38^. Parallel to this, hypergraph learning for multi-agent dynamics, implicit depth function estimation, and convolution-transformer hybrids have enriched computational modelling and feature extraction^39–41^. UAV-based geo-localisation, optical-ISAR image translation, and agricultural classification through deep models highlight applied AI in diverse domains^42–44^. Similarly, orthoimage generation, anomaly detection in hyperspectral images, and defect detection frameworks exemplify advances in remote sensing and industrial inspection^45–47^. Further, open-vocabulary dense prediction, adaptive visual odometry, and aerodynamic simulations emphasise real-time applications in robotics and sustainable design^48–50^. Ground extraction frameworks, aerial vehicle image segmentation, and health-oriented construction waste assessments extend the scope toward practical deployments^51–53^. Additionally, diffusion-based GANs for marine biology perception, perceptual fidelity estimation, and bio-inspired collision avoidance underline interdisciplinary integration^54–56^. Neuromorphic-inspired attention models, ISAR segmentation, and GAN-based image dehazing continue to push boundaries of visual intelligence^57–59^. Recent works have also explored 3D circle detection in point clouds, salient object detection, and collaborative transformers for continual learning, ensuring robustness and adaptability across domains^60–62^.

Moreover, it has also revolutionised RADP by elucidating land usage and the distribution of natural resources as per human activities in biological processes. Most of the traditional methods for LCS primarily relied on statistical and rule-based approaches^63^, which required extensive manual input and domain expertise. Techniques such as maximum likelihood classification^64^, K-means clustering^65^, and the iterative self-organising data analysis^66^ were commonly used to classify satellite imagery based on spectral properties. These traditional ML methods, like random forests (RF)^67^ and gradient machines, achieve accuracies of up to 80% on benchmark datasets. Moreover, the methods of ML-like Naïve Bayes, support vector machines (SVMs)^68^, and RF have also been utilised by the researchers to handle intricate landscapes and high-resolution data, posing difficulties. However, the level of segmentation and classification accuracy (70% to 77%) was compromised. Quantitative improvements, coupled with innovations like transfer learning and multi-modal data fusion, are expected to enhance further segmentation performance and its real-world applicability^69,70^. Recent advancements in DL, particularly CNNs and U-Net architectures, have transformed segmentation challenges by enhancing accuracy and generalisation. A study of the DeepGlobe Land Cover dataset revealed that DeepLabv3 + achieved an 88% mean Intersection over Union (mIoU), whereas conventional SVMs attained 72%^71^. Moreover, the Vision Transformers (ViT) achieve exceptional mIoU scores exceeding 90%, but it was first assisted with the pre-trained models and data augmentation^72^.

Ahmed. et al. Assessed Naïve Bayes classification^20^ applied to high-resolution aerial imagery obtained from a UAV-based RS. K-means clustering facilitates the selection of training and employs the method for linear and quadratic discriminant analyses with training set sizes ranging from 10 to 100 pixels. A training set size of 20 maximises classification accuracy and the Kappa coefficient up to an accuracy of 88.89% and 0.875, respectively. The size of the data was imbalanced as well as very small, compared to the dataset images available in the EuroSAT dataset^13^. In 2020, a DL-based approach was applied to limited labelled data to categorise high-resolution RS imagery; this geosystem approach addresses the issue by examining the genetic uniformity of spatially adjacent things across various sizes and hierarchies on the EuroSAT dataset classification with an accuracy of 91.52. However, an expanded training dataset was utilised in the training without the augmentation of the data, which was one of the limitations of that geosystem DL approach^21^. E. Kroupi et al. present a semiautomatic land-cover classification algorithm based on a deep CNN to address complex vision challenges and pose difficulties with an accuracy of 86% for the testing data. However, the accuracy was found to be degraded when exploited for noisy data. The approach encompasses elevated computational expenses, extensive labelled datasets, and challenges in generalising to the EuroSAT dataset^22^. Moreover, in 2025, J.A. Khan et al. exploited the same dataset along with two other datasets and presented a fuzzy-based deep CNN architecture to classify land cover and land sliding. The proposed architecture consists of 40 convolutional layers, 4 pooling layers, 4 inverted bottleneck blocks, 4 bottleneck blocks, and one fully connected layer to handle the effects of noise and inter-image interference^23^ with an accuracy of 93.3% with a quite high computational budget. From the comprehensive literature review in recent years, as presented in Table 3, there is still room for formulating a better, comprehensive, automated solution for land cover through a segmentation process with a high level of accuracy and other performance measures.

**Table 3:** State of the art related work.

Methodology	Accuracy (%)	References	Dataset and types
Gaussian naïve bayes	88.89	Ahmad et al. (2021)^20^	EuroSAT dataset^13^, publicly available
Deep CNN	86.00	Eleni Kroupi et al. (2019)^22^	
Geosystem approach	91.52	Yamashkin et al. (2020)^21^	
Fuzzy DL + chaotic PSO	93.30	J. A. Khan et al. (2025)^23^	

This paper presents a robust modified ViT that performs classification through segmentation by using the capabilities of the Squeeze and Excitation (SE) module with ViT. However, the proposed methodology is applied to a renowned public dataset called EuroSAT. The integration of SE modules after each transformer block dynamically recalibrates the channel-based feature maps. The global average pool layer is used to combine spatial information across each channel by using attention weights, scaling the feature maps, effectively expanding the most relevant channels and suppressing irrelevant ones. The optimization of the hyperparameters of the ViT-SE and feature selection procedure via an efficient global modified firefly algorithm that is designed by introducing inertia factor “$\psi$” and diversity in the standard firefly algorithm. The results are compared with state-of-the-art pre-trained models like VGG-19, ResNet-50, DarkNet-19, Inception-V3, and other reported contributions that exploit Gaussian Naïve Bayes, geosystem approach, deep CNN and fuzzy DL hybrid with chaotic PSO techniques on the same EuroSAT dataset. The stability as well reliability of the proposed architecture are validated through Monte Carlo simulations as well as statistical analysis of 200 independent runs.

## Material and methods

**Fig. 1:**
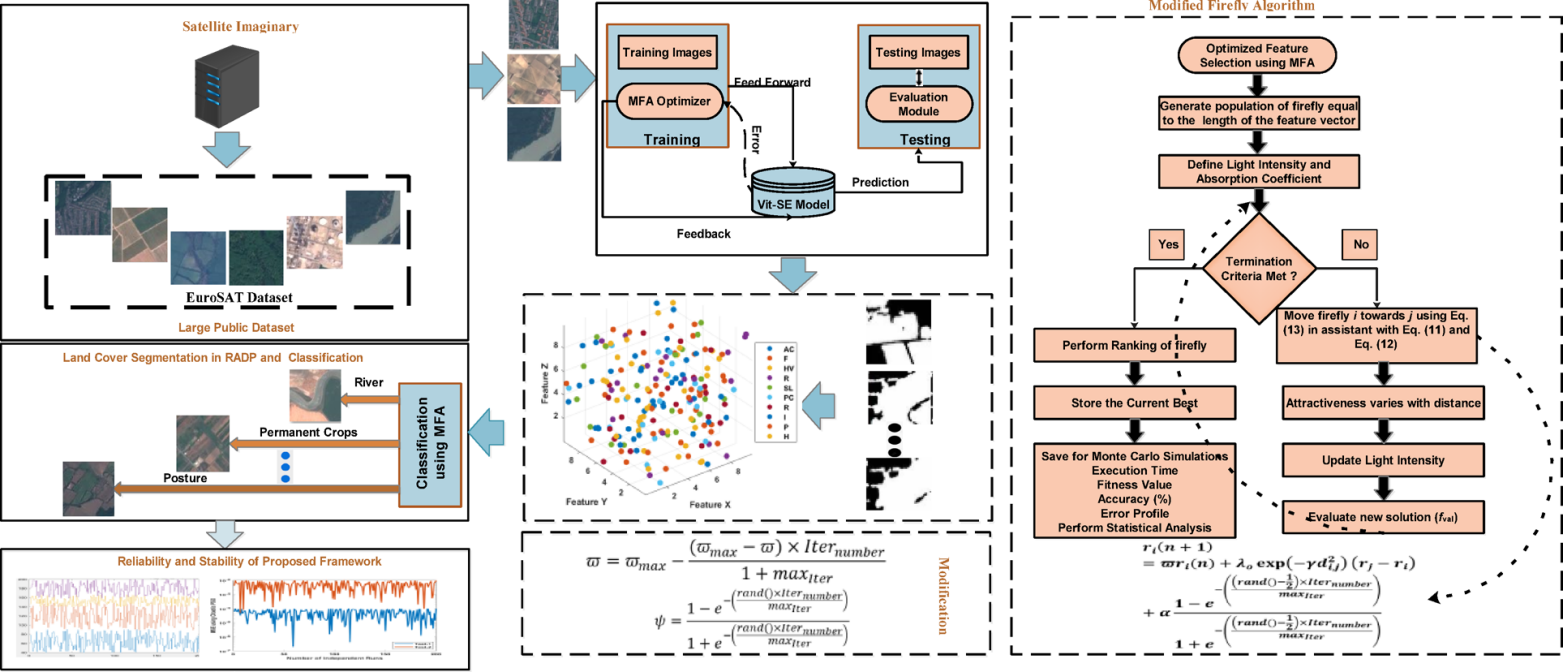
Graphical workflow of the proposed DL framework.

This section includes details of the dataset exploited along with the complexity of the RADP. The developed ViT-SE transformer and modified firefly algorithm, along with the pseudocode formulated, are also presented in this section. The graphical workflow of the proposed DL framework is presented below in Fig. 1.

### Details of the dataset

**Fig. 2:**
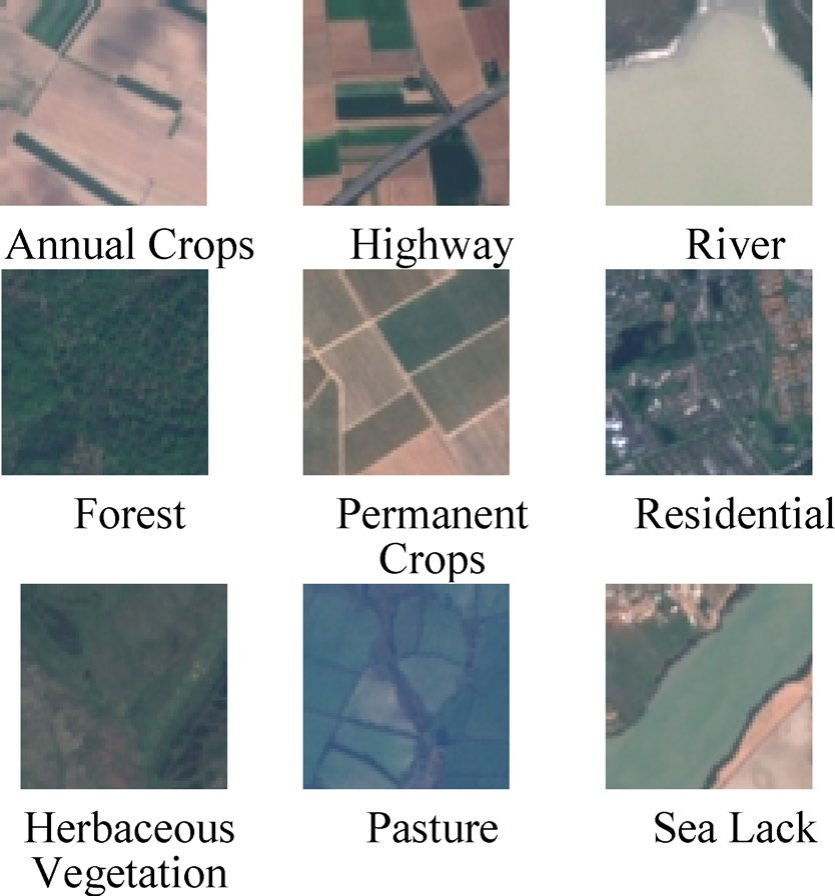
A few samples of the rural land cover dataset.

The dataset used as a benchmark is EuroSAT^13^ which is derived from Sentinel-2 satellite imagery in RGB format. Its 13 spectral bands, which contain 27,000 tagged and geo-referenced samples having 10 classes of land-covers with annual crop (AC), forest (F), herbaceous vegetation (HV), highway (H), industrial (I), pasture (P), permanent crops (PC), residential (Re), river (R) and sea lake (SL). The Classes AC, F, HV, R, PC, and SL have 3000 images each, while the classes Re and I contain 2500 images, respectively. Moreover, the Class of P and H have 2000 images. All the images have a bit depth of 24 and a size of 64 × 64 that cover a geographic area of approximately 6.4 km × 6.4 km on the ground. The nature of the dataset is complex; few of the classes have interference of feature segmentation and the size of the dataset is quite big and cannot be handled manually or by classical techniques as given in the literature survey. Given the high dimensionality of labelled classes, it is important to use it after feature selection and model training. Interference caused by AC, HV and PC classes causes feature optimization a challenging task. The balanced class distribution of the images ensures unbiased training and evaluation. Eurostat dataset preprocessing prepares Sentinel-2 satellite images for ML in numerous processes. First, raw Sentinel-2 multispectral photos with 13 spectral bands and 10m, 20m, and 60m spatial resolution are obtained. The dataset is divided into 64 × 64 pixel patches for each land-use class. Radiometric and geometric corrections improve image quality, including atmospheric haze and distortion removal. The whole 13-band dataset is kept for hyperspectral analysis; however, ordinary deep learning algorithms transform it to RGB. Standardizing pixel intensity levels by normalization ensures data distribution. Flipping, rotation, and contrast modifications improve the model’s robustness. Finally, each Class of the dataset is split into 80% for model training and 20% for evaluation of the model. EuroSAT, generated from Sentinel-2 satellite imagery, has 13 spectral bands that capture distinct wavelengths of light for land-use classification and remote sensing. The Blue (B2, 490 nm), Green (B3, 560 nm), and Red (B4, 665 nm) bands are essential for vegetation monitoring and water body analysis. In contrast, the Coastal Aerosol (B1, 443 nm) corrects the atmosphere Red Edge bands (B5, B6, B7) to identify plant stress and crop health, whereas Near-Infrared (NIR, B8, 842 nm) and Narrow NIR (B8a, 865 nm) improve biomass and vegetation classification^73^. The Water Vapor band (B9, 945 nm) corrects air conditions, whereas the Shortwave Infrared bands (SWIR1–B11 at 1610 nm, SWIR2–B12 at 2190 nm) detect soil moisture, drought, and land categorization. SWIR-Cirrus (B10, 1375 nm) detects high-altitude clouds. EuroSAT is a powerful dataset for multispectral image analysis since its 10m, 20m, and 60m bands enable accurate environmental monitoring, urban planning, agriculture analysis, and climate studies^74^. A few of the sample images of the classes of this rural dataset are shown in Fig. 2, which utilized satellite imagery from Sentinel-2, Landsat, and Planet Scope, which encompasses vast areas at a resolution of 10 m per pixel Moreover, an image resolution of 512 × 512 is used as an optimal balance for rural segmentation.

### Modified vision transformer

ViT^75^ is a powerful tool in medical imaging because it enables the processing of global spatial information through an attention mechanism^76^. However, its vanilla architecture often overlooks the interdependence of channel-wise fines critical to extracting detailed and meaningful features. To overcome these problems, the SE-based modified ViT is introduced with the name of ViT-SE Transformer, which integrates the SE modules after each transformer block. The SE module operates by dynamically recalibration of channel-based feature maps. After each transformer block, the SE mechanism applies the global average pool to combine spatial information across each channel of information and learn the importance of each channel. Then, using attention weights, scale the features maps, effectively expanding the most relevant channels and suppressing irrelevant ones.

The robust ViT-SE constantly readjusts feature representations to enhance segmentation and classification. It adeptly captures global dependencies and selectively enhances spatial and channel-wise attributes. It also enhances object boundary precision and classification accuracy by emphasizing discriminative features, rendering it beneficial for semantic an instance segmentation. ViT-SE is an effective solution for complex computer vision applications, demonstrating excellence in pixel-wise segmentation and image-level classification due to its customizable focus.

The proposed ViT-SE transformer accepts the input of 384 × 384 × 3. The initial layer is patch embedding is employed with 16 × 16 patch sizes and applies position encoding to retain the spatial information. This positional encoding is added element-wise to the patches and is represented as:

1 $$\left. {\begin{array}{*{20}c} {{\mathcal{T}}_{0} = {\mathcal{T}}_{0} + P_{n} } \\ {P_{n} \left[ k \right] = \sin \left( {\omega .k} \right),{\text{cos}}\left( {\omega ,k} \right)\left( {P_{f} ,\omega } \right)} \\ \end{array} } \right\}$$

where ${\mathcal{T}}_{0}$ is the outcome of the positional encoding and ${P}_{f}$ is the predefined frequency. The first Transformer is started by employing multi-headed self-attention (MFA) layer with 3 heads to perform self-attention across each patch in order to learn the long-range relationship whose mathematical relationship is given in (2).

2 $$\varphi_{A} \left( {\alpha ,\beta ,\delta } \right) = Softmax\left( {\frac{{\alpha \beta^{\tau } }}{{\sqrt {d_{h} } }}} \right)\delta ,\varphi_{MHSA} \left( {\mathcal{T}} \right) = \oplus \left( {h_{1} ,h_{2} ,h_{3} } \right)W_{0} , W_{0} \in {\mathbb{R}}^{{\left( {h.d_{h} } \right) \times d}}$$

where ${\varphi }_{MHSA}$ is the outcome of MHSA. After that, a residual connection is added, followed by the normalization layer that is defined as:

3 $${\mathcal{T}}^{\prime}=N({\varphi }_{MHSA}\left(\mathcal{T}\right)+\mathcal{T})$$

**Fig. 3:**
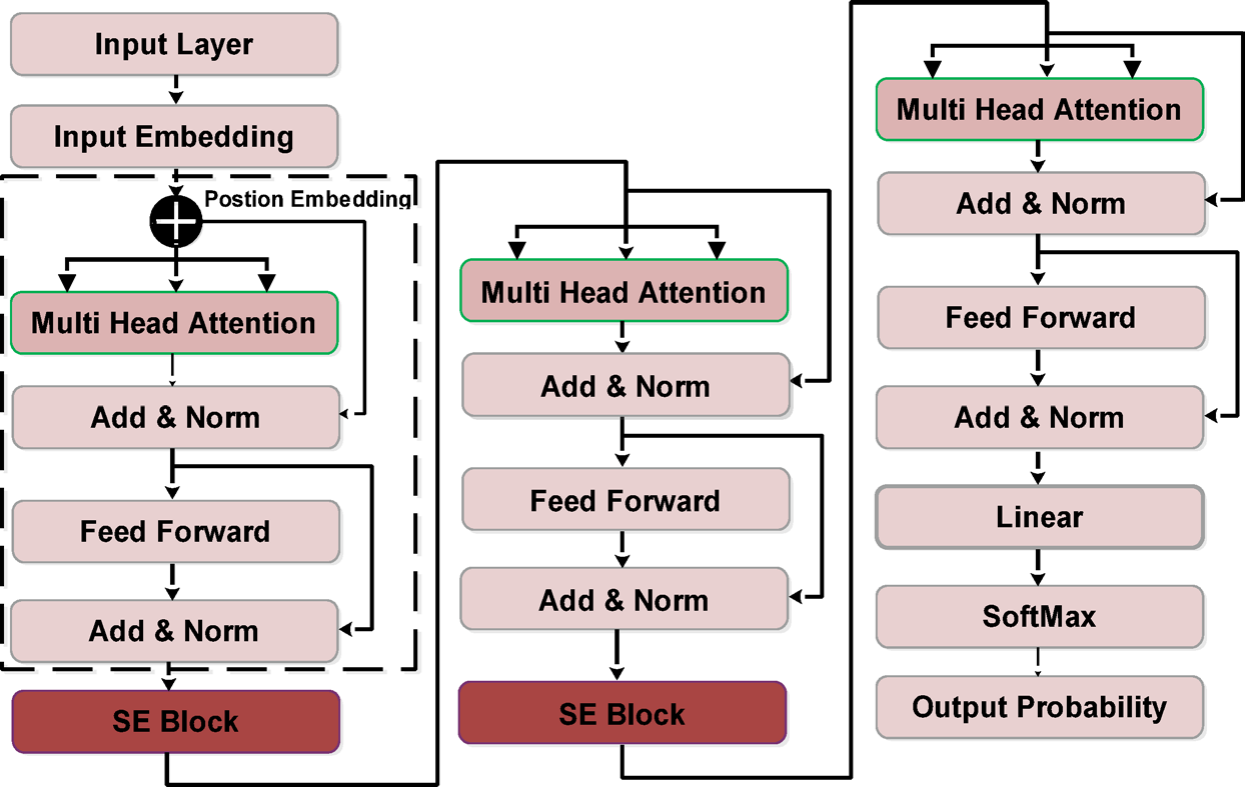
Proposed ViT-SE transformer model.

In addition, the FFN process is performed on the outcome of the MHSA layer. This process consists of two linear layers with nonlinear activation. We have our FFN layer is:

4 $${\varphi }_{FFN}=\phi \left(\mathcal{T}{w}_{1}+{b}_{1}\right){w}_{2}+{b}_{2}, {w}_{1}\epsilon {\mathbb{R}}^{d\times {d}_{f}} and {w}_{2}\epsilon {\mathbb{R}}^{{d}_{f}\times d}$$

where $\phi$ is the GeLU activation, after the first Transformer, the SE block is performed to apply channel-wise attention to the feature maps for enhancing the most relevant features. The SE module contains the global average pooling, fully connected and sigmoid activation. The mathematical modeling of the SE block is defined in (5) as follows:

5 $$\varphi_{SE} = \left\{ {\begin{array}{*{20}l} {g_{p} = \frac{1}{H,W}\mathop \sum \limits_{a = 1}^{H} \mathop \sum \limits_{b = 1}^{W} {\mathcal{T}}_{a,b,s} } \hfill \\ {f_{1} = \sigma \left( {w_{2} .\emptyset \left( {w_{1} .s} \right)} \right)} \hfill \\ {{\mathcal{T}}_{a,b,p} .f_{c} } \hfill \\ \end{array} } \right.$$

where ${g}_{p}$ is the global average pooling operation, and $H,W$ is the height and width of the feature map, ${f}_{1}$ is the fully connected operation, $s$ is the squeezed value of the channel. ${w}_{1},{w}_{2}$ is the weight matrices of the fully connected layer, $\sigma , \varnothing$ is the sigmoid and ReLU activation, respectively.${\mathcal{T}}_{a,b,p}$ is the recalibrated activation and ${f}_{c}$ is the excitation weights of channels. After this, one more two transformer block is added with the SE block as described above. In the end, a cls-indexing layer was added to capture the global representation and then employed classification head by using Eq. (6).

6 $$\varphi_{SE} = \left\{ {\begin{array}{*{20}l} {{\mathcal{T}}_{CLS} = T\left[ 0 \right]} \hfill \\ {{\mathcal{T}}_{FC} = W.{\mathcal{T}}_{CLS} + b} \hfill \\ {C_{k} = \frac{{e^{{{\mathcal{T}}_{k} }} }}{{\mathop \sum \nolimits_{x = 1}^{K} e^{{{\mathcal{T}}_{k} }} }}, k \in \left[ {1,K} \right]} \hfill \\ \end{array} } \right.$$

where ${\mathcal{T}}_{CLS}$ is the outcome of the cls-indexing layer, ${\mathcal{T}}_{FC}$ is the fully connected layer that passed to the softmax activation for the final output ${C}_{k}$. The high-level architecture is shown in Fig. 3.

In ViT-SE architecture, the self-attention mechanism is successively integrated with the SE module to enhance the model’s representational capacity. Initially, the input image is divided into fixed-size patches, which are then linearly embedded and passed into the Transformer encoder. Within each encoder block, the MHSA mechanism is applied to capture long-range dependencies and spatial relationships among patches, followed by an FFN for further feature transformation. After this, the output is passed through the SE module, which performs channel-wise recalibration. The SE module first applies global average pooling to compress spatial dimensions into a compact channel descriptor, then uses a small feedforward network to learn channel-wise dependencies and generate attention weights. These weights are used to scale the output feature maps, emphasizing more informative channels and suppressing less useful ones. By combining the spatial attention of the Transformer with the channel attention of the SE module, the ViT-SE architecture effectively captures both global contextual relationships and fine-grained channel importance, leading to improved performance in visual recognition tasks. Moreover, it is worth mentioning that the SE module and Transformer encoder layer are systematically combined to enhance feature recalibration and representation learning. The SE module adaptively recalibrates feature responses on a channel-wise basis to enable the model to prioritize salient information hile diminishing the significance of less important data. This hybrid architecture utilizes the self-attention mechanisms of the Transformer encoder to record long-range dependencies and contextual information. The SE module enhances input feature maps prior to or subsequent to the attention mechanism, enabling the Transformer to utilize supplementary discriminative characteristics.

### Modified firefly algorithm

The standard firefly algorithm was developed by Xin-She Yang^76^ that is a nature-inspired optimization technique based on the social behavior and bioluminescent communication of fireflies. It has been applied in every domain of science and engineering to handle infinite space and multi-dimensional complex problems. It simulates how fireflies are attracted to brighter individuals, with brightness representing the fitness of a solution in optimization problems. The algorithm uses a combination of deterministic movement towards brighter fireflies and random exploration to find global optima. The attractiveness of a firefly decreases with distance, governed by a light absorption coefficient, which balances exploration and exploitation. The light intensity “$\delta$” of the firefly is represented (7):

7 $$\delta (d)=\frac{{\delta }_{o}}{1+\gamma {d}^{2}}$$

where $\delta (d)$ intensity of light of the firefly is dependent on the distance between two fireflies and $\gamma$ absorption coefficient. Similarly, the attractiveness is defined by the relation in (8).

8 $$\lambda (d)=\frac{{\lambda }_{o}}{1+\gamma {d}^{2}}$$

where ${\lambda }_{o}$ is the highest brightness of the firefly that is dependent on the distance between the firefly “$d$”. The distance between two fireflies *i* and *j,* at ${r}_{i}$ and ${r}_{j}$ is the cartesian distance between them that is given in (9)

9 $${d}_{i,j}=\Vert {r}_{i}-{r}_{j}\Vert =sqrt\left(\sum_{k=1}^{D}{\left({r}_{i}-{r}_{j}\right)}^{2}\right)$$

The movement towards the brighter firefly “*j*” by the firefly.

“*i*” is determined by the standard equation of the firefly algorithm as given below:

10 $${r}_{i}\left(n+1\right)={r}_{i}(n)+{\lambda }_{o}\text{exp}\left(-\gamma {d}_{i,j}^{2}\right)\left({r}_{j}-{r}_{i}\right)+\alpha (rand\left(\right)-\frac{1}{2})$$

#### ***Modification: the inclusion of inertia factor “***$\boldsymbol{\varpi }$***”***

The modification proposed in the standard algorithm is the inclusion of the inertia factor $\varpi$ as given in (11) that defines the thrust in the current position of the firefly during the position updating process.

11 $$\varpi ={\varpi }_{max}-\frac{{(\varpi }_{max}-\varpi ){\times Iter}_{number}}{1+{max}_{Iter}}$$

where ${\varpi }_{max}$ and ${\varpi }_{min}$ are the maximum and minimum values of the inertia factor.

#### Modification: inclusion of geometric factor “ψ”

Similarly, a geometric factor “$\psi$” is also introduced that is adjusted by a controlled space random value operated by tan-sigmoid function as provided in (12).

12 $$\psi =\frac{1-{e}^{-\left(\frac{rand(){\times Iter}_{number}}{{max}_{Iter}}\right)}}{1+{e}^{-\left(\frac{rand(){\times Iter}_{number}}{{max}_{Iter}}\right)}}$$

Therefore, the relation after modification is observed to be more exploratory and diverse; its relationship is provided in (13)

13 $${r}_{i}\left(n+1\right)=\varpi {r}_{i}(n)+{\lambda }_{o}\text{exp}\left(-\gamma {d}_{i,j}^{2}\right)\left({r}_{j}-{r}_{i}\right)+\alpha \frac{1-{e}^{-\left(\frac{\left(rand()-\frac{1}{2}\right){\times Iter}_{number}}{{max}_{Iter}}\right)}}{1+{e}^{-\left(\frac{\left(rand()-\frac{1}{2}\right){\times Iter}_{number}}{{max}_{Iter}}\right)}}$$

### Performance measures and statistical parameters

The performance measures used to see the quality of the segmented regions through the proposed architecture at the pixel level are taken as overall accuracy (%) and kappa coefficient. Moreover, the class-specific metrics exploited are precision, recall and F1 score by using the standard mathematical relation well known in the literature^77^. IOU is utilized to see the over-segmentation and under-segmentation, and average IoU across all the classes^78^. The stability of the proposed framework is obtained through Monte Carlo simulation for 200 independent runs and their statistical investigations based on standard statistical operators. This analysis is based on the Minimum value of the accuracy achieved “Min”, maximum value of the accuracy “Max”, mean ($\overline{\mu }$), standard deviation ($\sigma$) and kurtosis ($\widehat{\kappa }$) and kappa coefficient ($\Xi$)^79^. The mathematical formulas^80^ for $\mu$, $\sigma$, $\widehat{\kappa }$ and $\Xi$ are given in Eqs. (14), (15), (16), and (17), respectively.

14 $$\mu =\frac{1}{N}\sum_{i=1}^{N}{z}_{i}$$

15 $$\sigma =\sqrt{\frac{1}{N}\sum_{i=1}^{N}{\left({z}_{i}-\mu \right)}^{2}}$$

16 $$\widehat{\kappa }=\frac{N(N+1)}{(N-1)(N-2)(N-3)}\sum_{i=1}^{N}{\left(\frac{{z}_{i}-\mu }{\sigma }\right)}^{4}-\frac{3{(N-1)}^{2}}{(N-2)(N-3)}$$

17 $$\Xi =\frac{{P}_{o}-{P}_{e}}{1-{P}_{e}}$$

$$where {P}_{o}= \frac{TP+TN}{TP+TN+FP+FN} and$$

$${P}_{e}= \left(\frac{\left(TP+FP\right)\left(TP+FN\right)+\left(FN+TN\right)\left(FP+TN\right)}{{\left(TP+TN+FP+FN\right)}^{2}}\right)$$

where ${z}_{i}$ is stored result in each independent run, and N is the total number of results stored for accuracy, precision, recall and F1 score.

**Table:** 

**Training Protocol for the Proposed ViT-SE + MFA Framework** **Step 1: Initialization** • Initialize a random population “${p}_{n\times l}$” of $n=50$ fireflies, where each firefly length ll equals the length of the feature vector. • Position fireflies within the search space. • Set the MFA parameters as given in Table 5 for $\xi$, $\gamma$*,* ${\lambda }_{o}$, α and max_*Iter*_, respectively. **Step 2: Objective Function Creation** • Define the objective function $f(p$) to optimize feature selection for the ViT-SE extracted features. • Assign initial light intensity δ to each firefly, representing its brightness (solution quality). **Step 3: Firefly Movement & Exploration** • For each firefly *i*, compare brightness δ_*i*_ with every other firefly j. • If ${\delta }_{i}>{\delta }_{i}$ , move firefly ii toward j; otherwise, perform a random walk for exploration. • Compute distance dd using relation (9). • Calculate attractiveness λ via relation (8) and update position using the modified inertial and geometric factors in relation (13). • Recalculate fitness and update brightness for each firefly. **Step 4: Ranking & Selection** • Rank all fireflies by brightness using index sort and retain the best solutions. • Gradually reduce the randomization factor over iterations to transition from exploration to exploitation, avoiding premature convergence. • Repeat Steps 3 and 4 until termination conditions are met. **Step 5: Termination Criteria** • Maximum iterations reached. • Features optimized to best subset. • No improvement over 10 consecutive iterations. **Step 6: Monte Carlo Simulation & Statistical Analysis** • Perform 200 independent runs. • Compute statistical measures $\mu$, $\sigma$, $\widehat{\kappa }$ and $\Xi$ from relations (14)–(17).	

## Results and discussion

The proposed architecture based on ViT-SE and MFA is validated on a standard benchmark dataset of EuroSAT, whose classification is consistent with real-world rural geography. In this regard the proposed framework is helpful for RADP. The proposed framework is not only compared with state-of-the-art pre-trained models like VGG19, ResNet50 and Inception V3 but also the reported scheme provided in Table 3. The simulations are performed using MATLAB® version R2023b on an Intel (R) Core processor i9, 64 GB of RAM, and an 8 GB NVIDIA RTX graphics card. The dataset is split randomly into a ratio of 80:20 in such a way that 80% of the images of each Class are used as training while 20% remain blind for validation of the proposed framework for testing. An input of 384 × 384 × 3 is passed to the ViT-SE transformer with a patch size of 16 × 16 and a dropout rate of 0.2. The embedding dimension is set as 1024 with 24 layers. Moreover the number of attention heads is obtained as 16 with a feedforward dimension of 4096 as hyperparameters optimized by MFA. The learning rate $\alpha$ is set to be 0.0001 however, the behavior of the framework is also observed by varying $\alpha$ from 0.01 to 0.0001 with a fixed epoch of 100. The hyperparameter values of the proposed DL model have been tabulated in Table 4, while the parameter values and setting used for the simulation purpose for feature selection through MFA are provided in Table 5.

**Table 4:** Hyperparameter values for the proposed framework.

Hyperparameters	Values
Input size	384 × 384 × 3
Patch size	16 × 16
Dropout rate	0.2
Embedding dimension	1024
feedforward dimension	4096
Attention head	16
Optimizer	MFA
Split ratio	80 (training): 20 (testing)
Other	Default

**Table 5:** Parameter values and their settings used in simulations.

Parameter values/setting	
Parameters	Values
Swarm size	50
Max. iterations	700
$\xi$	[0.0001 to 0.01]
$\gamma$	5
${\lambda }_{o}$	0.4
α	0.01
Distance metric	Euclidean distance
*f*val	10^–12^
Scaling factor	Random
Other options	Default

A scenario-based simulation has been performed on the EuroSAT dataset by applying the parameter values and setting given in Table 5 on the performance measure listed in the previous section. The results in the form of a confusion matrix are presented in Table 6 after applying the proposed framework. The table illustrates the performance of the classification model in a multi-class scenario comprising 10 categories that exhibit categorization accuracy of over 99%. The model precisely recognized 99.5% (2388 samples) AC, 99.375% (2385 samples) F, and 99.667% (2392 samples) HV, while the misclassifications are infrequent within the matrix. It is quite evident from the table that 0.041% (1 sample) of AC was erroneously categorized as F, R, PC, and H. Moreover, F exhibited a 0.123% misclassification rate in AC over three samples, with modest errors distributed among the classes. P (Pasture) exhibited a misclassification rate of 0.1875% (3 samples) as SL, along with minor inaccuracies in other categories. Nonetheless, these mistakes are negligible and do not significantly impact model reliability. The matrix indicates that the model effectively distinguishes between similar classes, such as AC and PC or F and HV. The model demonstrates proficiency in land-cover classification, evidenced by its accuracy and few off-diagonal values, rendering it a viable choice for RADP.

**Table 6:** Confusion matrix of EurSAT dataset using the proposed framework.

True class	Predicted class (%)									
	AC	F	HV	R	PC	SL	Re	I	P	H
AC	**99.5** **(2388)**	0.041 (1)	0	0.041 (1)	0.082 (2)	0.082 (2)	0.041 (1)	0.082 (2)	0.041 (1)	0.082 (2)
F	0.123 (3)	**99.375** **(2385)**	0.082 (2)	0.041 (1)	0	0	0.205 (5)	0.082 (2)	0	0.082 (2)
HV	0.041 (1)	0.082 (2)	**99.667** **(2392)**	0	0	0.041 (1)	0.082 (2)	0	0	0.082 (2)
R	0.041 (1)	0.041 (1)	0.082 (2)	**99.458** **(2387)**	0.041 (1)	0.123 (3)	0.082 (2)	0.041 (1)	0.082 (2)	0
PC	0	0.123 (3)	0.082 (2)	0	**99.5** **(2388)**	0	0.123 (3)	0.082 (2)	0	0.082 (2)
SL	0.082 (2)	0.041 (1)	0	0	0.041 (1)	**99.58** **(2390)**	0.082 (2)	0	0.041 (1)	0.123 (3)
Re	0.05 (1)	0.05 (1)	0	0.1 (2)	0	0.05 (1)	**99.6** **(1992)**	0.1 (2)	0	0.05 (1)
I	0.1 (2)	0	0.1 (2)	0.05 (1)	0	0	0.05 (1)	**99.5** **(1990)**	0.1 (2)	0.1 (2)
P	0.125 (2)	0.0625 (1)	0.0625 (1)	0	0	0.1875 (3)	0	0	**99.5** **(1592)**	0.0625 (1)
H	0.0625 (1)	0	0.125 (2)	0	0	0.0625 (1)	0	0	0.125 (2)	**99.625** **(1594)**

Significant values are in bold.

**Fig. 4:**
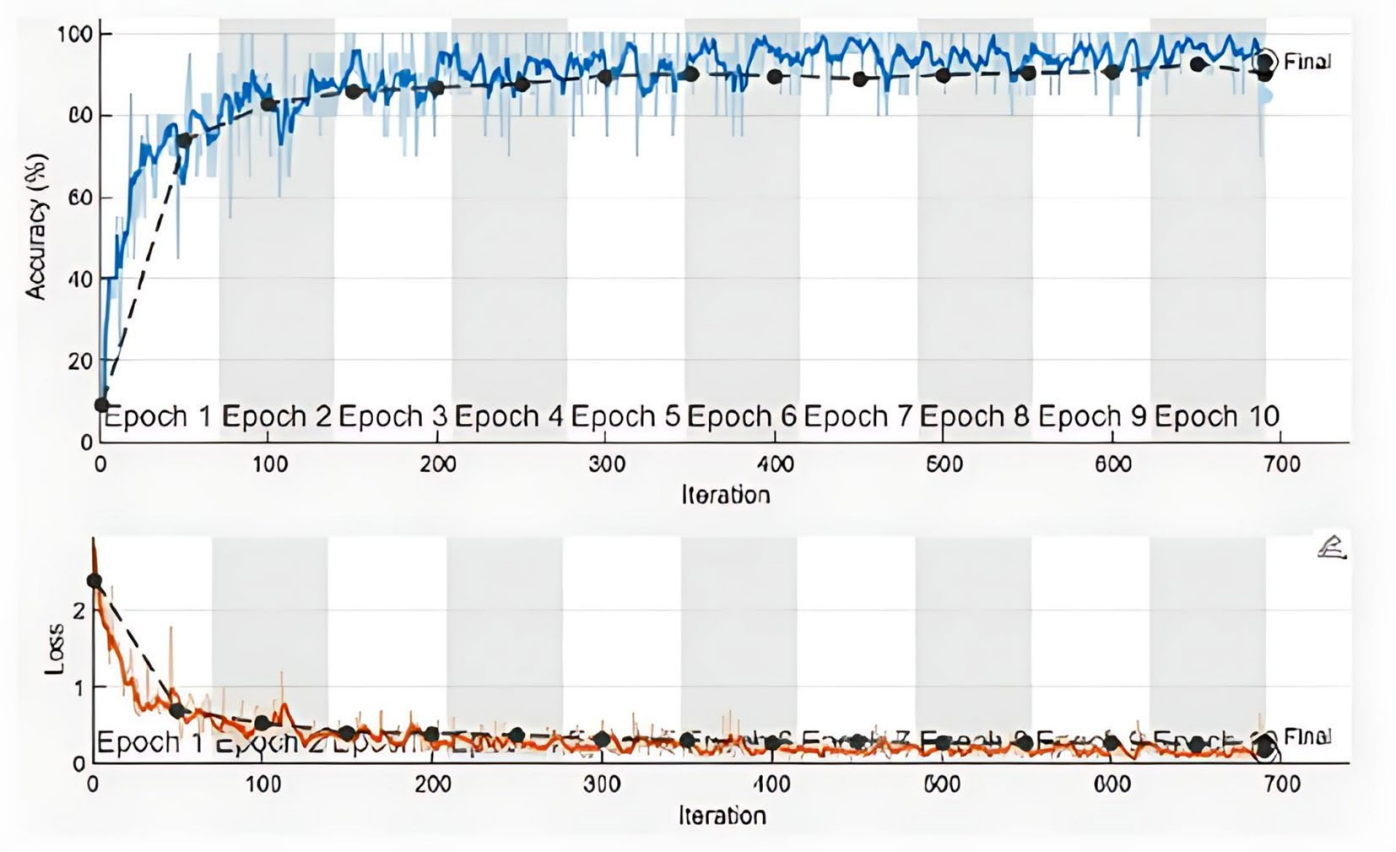
Training and loss function profile of the proposed architecture.

The overall accuracy of the model is found to be 99.53%, with a misclassification rate of 0.47% & 0.9953 weighted F1 score. The training percentage accuracy and loss function profile with 700 iterations and 10 epochs are drawn in Fig. 4. it is quite evident from the figure that as training advances, accuracy consistently improves, nearing 100% by completion, while training and validation loss is approaching zero. Initial accuracy surges indicate model learning, succeeded by stabilization as it converges. The validation accuracy closely aligns with the training accuracy, suggesting slight overfitting and robust generalization.

The loss decreases markedly during the initial epochs, signifying that the model rapidly optimizes its parameters. The loss diminishes gradually throughout training, stabilizing in the latter epochs. The proximity of the training and validation loss curves demonstrate the model’s resilience and its capacity for effective learning without overfitting.

The performance of the proposed model is also validated on performance measures like precision, recall, and F1-score of each recognition class and its results are tabulated in Table 7. It can be depicted from the table that the F1-scores of most classes, including HV (0.9960), R (0.9962), and PC (0.9967), demonstrate a balance between precision and recall. The PC achieves the best F1-score of 0.9967, signifying its precision and dependability. Class Re exhibits a recall of 0.9920 and an F1-score of 0.9940, both of which are slightly lower. H has the lowest recall (0.9907), which adversely impacts its F1-score (0.9935). Notwithstanding these slight modifications, the model’s accuracy across all categories varies from 0.9938 to 0.9967, illustrating its capacity to minimize false positives. The table indicates that the model exhibits consistent performance across all recognition categories with minimal fluctuation.

**Table 7:** Proposed architecture results for various performance measures.

Recognition class	Performance measure (classification)			Performance measure (segmentation)	
	Precision	Recall	F1-score	mIOU	Dice
AC	0.9950	0.9946	0.9948	0.9632	0.9812
F	0.9938	0.9958	0.9948	0.9624	0.9808
HV	0.9967	0.9954	0.9960	0.9612	0.9802
R	0.9946	0.9979	0.9962	0.9683	0.9838
PC	0.9950	0.9983	0.9967	0.9685	0.9839
SL	0.9958	0.9954	0.9956	0.9672	0.9833
Re	0.9960	0.9920	0.9940	0.9629	0.9810
I	0.9950	0.9955	0.9952	0.9648	0.9821
P	0.9950	0.9950	0.9950	0.9647	0.9820
H	0.9962	0.9907	0.9935	0.9626	0.9809

**Fig. 5:**
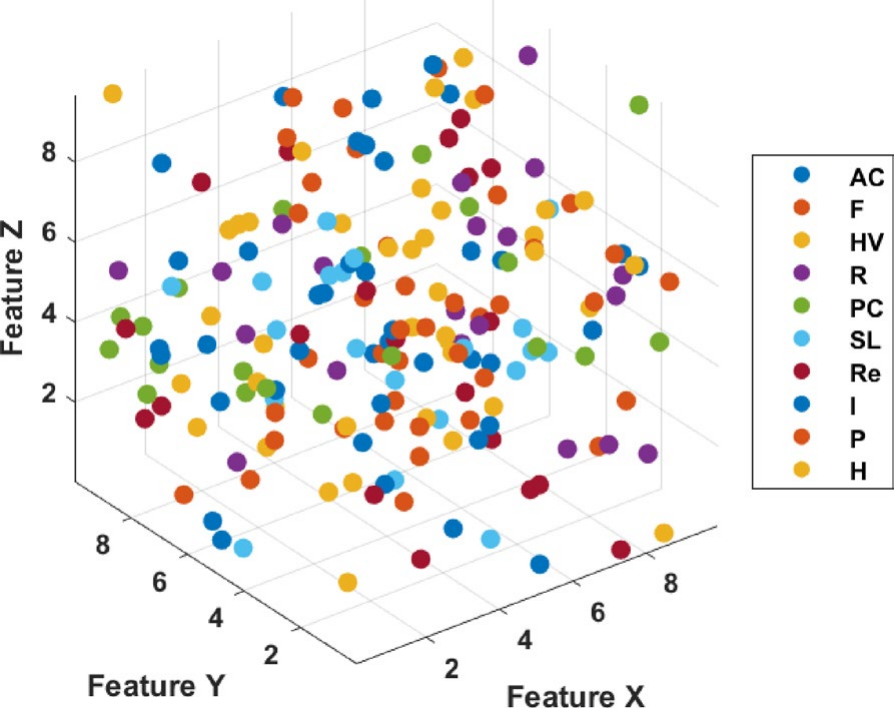
3D scattered plot for feature distribution for categorization.

To see the insight strength of the proposed ViT-SE, the 3D scatter plot for the distribution of features across multiple classes is presented in Fig. 5, a multi-class dataset.

**Fig. 6:**
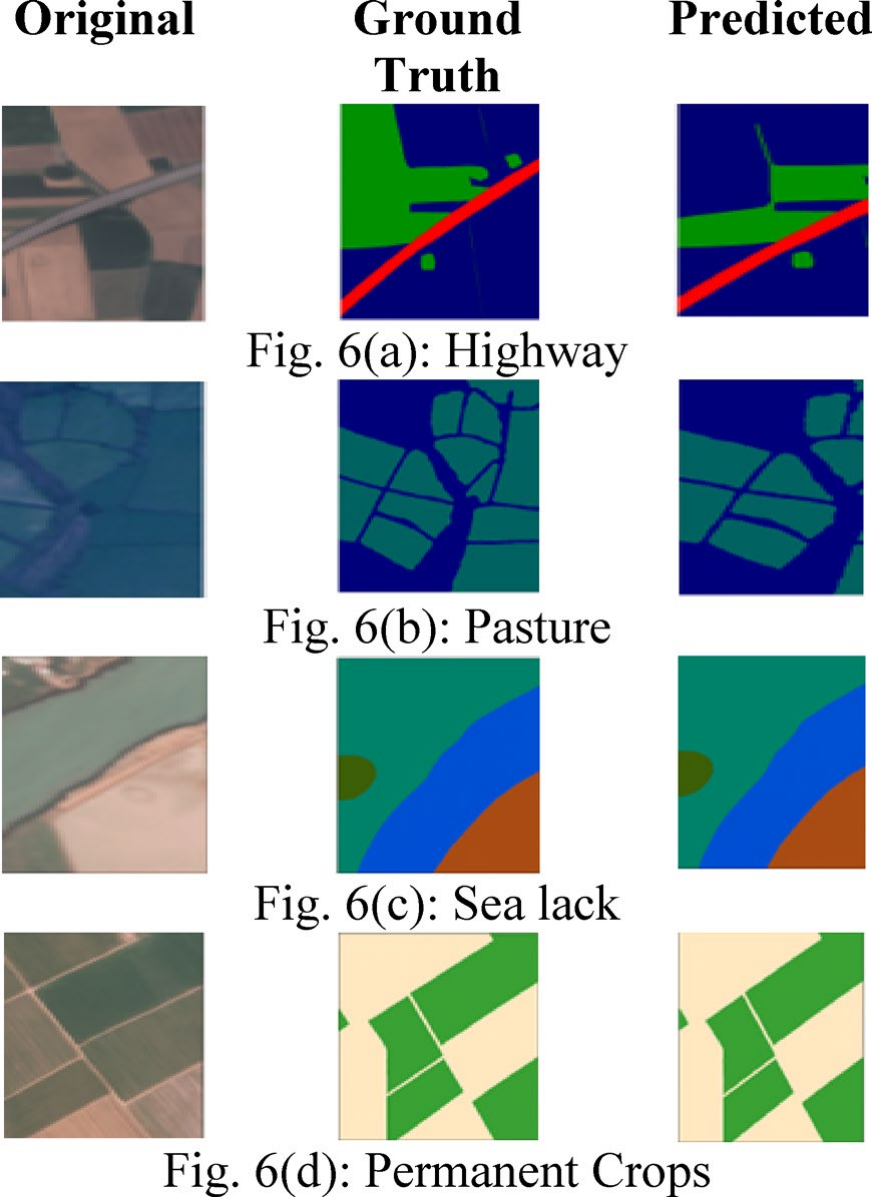
Qualitative segmentation results of the model on different types of land covers. (**a**): Highway. (**b**): Pasture. (**c**): Sea lack. (**d**): Permanent Crops.

The axes Feature X, Feature Y, and Feature Z denote three dimensions of the feature space that delineate the location of each plot point, which signifies a data instance and overlap. The figure indicates that the features somewhat distinguish the classes. However, further processing via MFA fine-tune the categorization of distinct classes throughout the space, signifying the diversity of the dataset’s feature distribution. Figure 6 presents qualitative segmentation results of the proposed architecture on different types of land covers, including highway (Fig. 6a), pasture (Fig. 6b), sea lack (Fig. 6c) and permanent crops (Fig. 6d), respectively. The figure presents the original, ground truth, and predicted images to visualize the semantic segmentation performance of a model in a rural region. The original image of Fig. 6c displays a satellite view of a water body, possibly a river or lake, with adjacent land cover, including vegetation and soil. The ground truth image shows manually annotated land cover classes, with distinct colours indicating different regions such as water, vegetation, and soil. The predicted image depicts the output of the segmentation model, which closely mirrors the ground truth with well-preserved boundaries between classes. The segmentation overlay demonstrates the model’s strong ability to distinguish between water bodies, highways, crops and surrounding land features. Minor discrepancies, such as slight shifts in boundaries or variations in the shape of certain regions, are observable but minimal, reflecting a high level of accuracy and spatial consistency in the model’s predictions.

### Ablation study 1: impact of the varying learning rates on performance measures

The impact of the efficiency and efficacy of the proposed model is examined by taking different values of the learning rates “$\xi$”. In this regard, the results are tabulated in Table 8, which utilizes criteria like accuracy, precision, recall, and F1-score. The findings indicate that decreasing the learning rate improves model efficacy. The model attains an accuracy of 98.09%, with precision at 0.9735, recall at 0.9754, and an F1-score of 0.9789, utilizing an elevated ξ = 0.01. As the learning rate diminishes to ξ = 0.013 and ξ = 0.001, accuracy enhances to 98.43% and 99.20%, respectively, accompanied by improved precision, recall, and F1-score. The model exhibits optimal performance at ξ = 0.0001 by achieving 99.50% accuracy, 0.9919 precision, 0.9912 recall, and 0.9953 F1-score, respectively. Reduced learning rates facilitate model convergence and enhance the precision of data pattern recognition. This trend is seen in the F1-score, which equilibrates precision and recall, achieving its peak value at ξ = 0.0001; therefore, reduced learning rates enhance model performance across all metrics, as illustrated in the table.

**Table 8:** Effect on accuracy with change in learning rate.

Learning rate ($\xi$)	Performance measures			
	Accuracy (%)	Precision	Recall	F1-score
0.01	98.09	0.9735	0.9754	0.9789
0.013	98.43	0.9789	0.9837	0.9817
0.001	99.20	0.9834	0.9896	0.9914
0.0047	99.37	0.9893	0.9905	0.9927
**0.0001**	**99.50**	**0.9919**	**0.9912**	**0.9953**

Significant values are in bold.

### Ablation study 2: monitoring the optimizer “MFA”

**Fig. 7:**
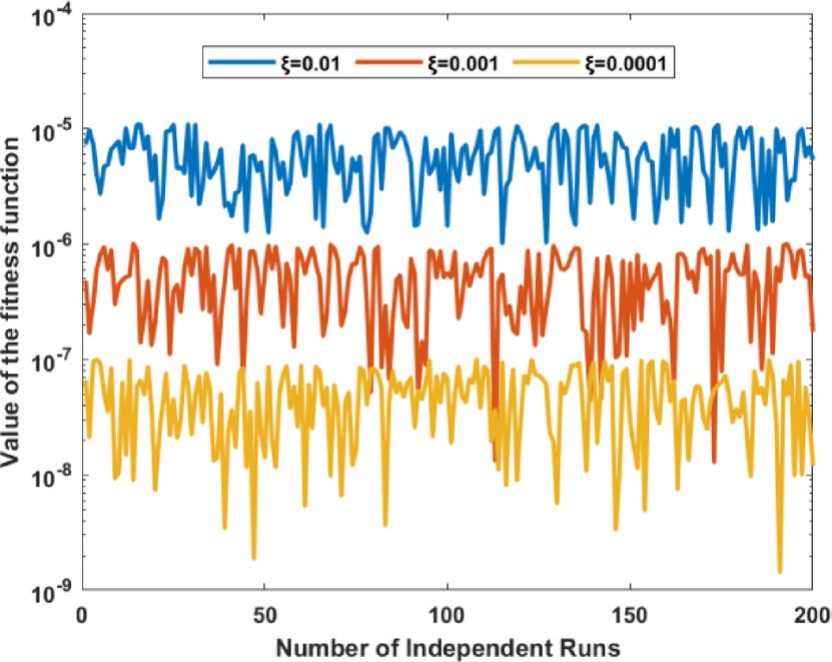
Proposed MFA behavior for 200 independent runs.

The capability of MFA is monitored by performing a cumbersome process of repetition for the evaluation of the fitness results in 200 independent runs, and results are plotted in Fig. 7 on a semiology scale to see a clear difference. It is quite clear from the graph that a fitness value between 10^–5^ to 10^–6^, 10^–6^ to 10^–7^ and 10^–7^ to 10^–9^ is observed for a learning rate of ξ = 0.01, ξ = 0.001 and ξ = 0.0001, respectively that depicts the efficacy of an optimization algorithm throughout 200 distinct trials. It is worth mentioning from the figure that MFA fitness values are highly sensitive to ξ and stabilize with minimal fluctuation.

The pattern indicates that reduced ξ values improve the optimization algorithm’s convergence to superior solutions by balancing exploration and exploitation. Enhanced stability at reduced ξ may indicate a compromise between solution quality and convergence velocity. This analysis demonstrates that enhancing algorithm performance necessitates the calibration of ξ.

### Ablation study 3: computational complicity in terms of time

**Fig. 8:**
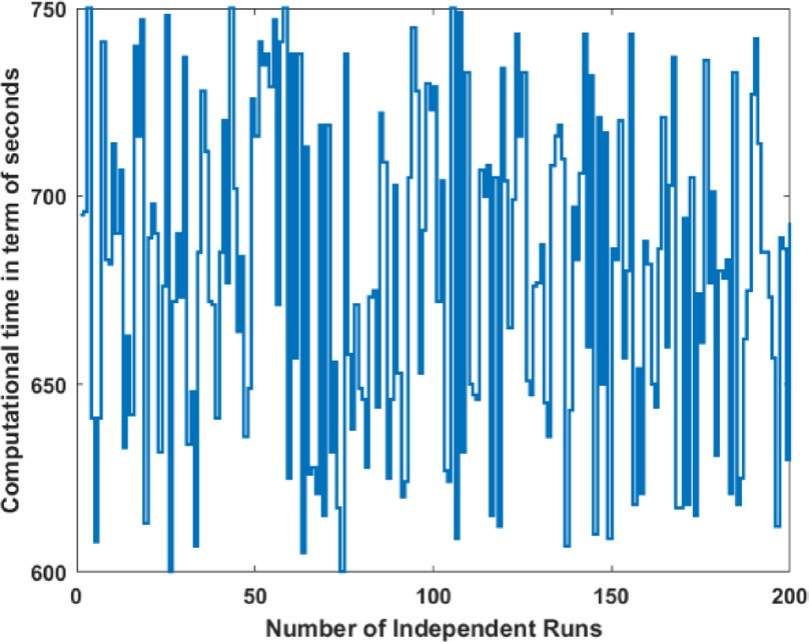
Computational load in seconds for each independent run.

It is difficult to find the space complexity due to the heuristic nature of the DL. However the computational time in seconds by the proposed model is illustrated in Fig. 8 for 200 algorithm executions. The analysis indicates that computing time fluctuates between 600 and 750 s for each execution.

This variation indicates that data complexity, operational conditions, and computational environment influence processing time. The majority of runs remain stable, while some increase, signifying elevated processing requirements. This may result from varying convergence behaviours or algorithmic approaches to certain conditions.

The graph illustrates the algorithm’s performance consistency and can assist in optimizing runtime variability and efficiency. It underscores the importance of computing demand analysis for efficient deployment, particularly for large-scale or time-critical applications. It is also worth mentioning that the mean execution time (MET) of 678.91 s is observed with a deviation of 42.574 s.

### Ablation study 4: reliability of the proposed model

**Fig. 9:**
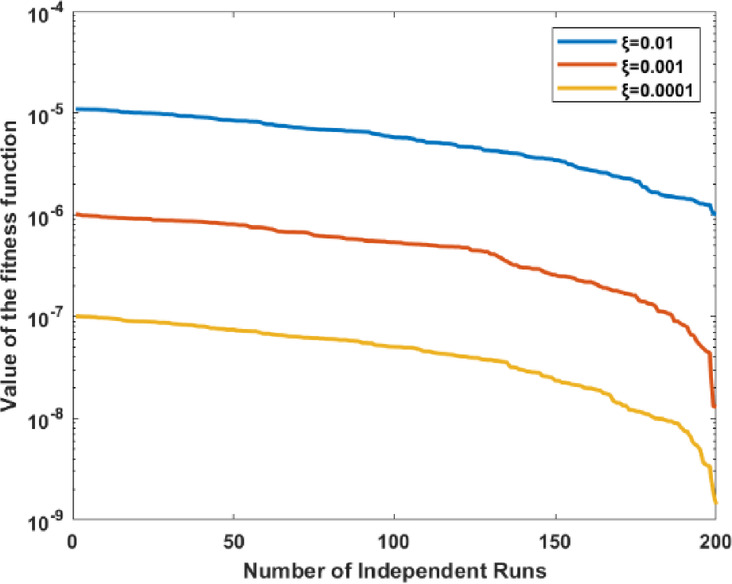
Proposed MFA behavior for 200 independent runs.

200 separate iterations investigate the reliability of the proposed models; the graph in Fig. 9 illustrates the convergence of the fitness function for three specific parameter values ξ = 0.01, 0.001 and 0.0001, respectively.

The x-axis represents the number of independent runs. At the same time, the y-axis denotes the value of the fitness function on a logarithmic scale to clearly demonstrate the superiority of optimization performance as well as consistent convergence.

In every case, the fitness function consistently decreases as the number of iterations increases, indicating an enhancement in solution quality over time. This graph emphasizes the need to adjust ξ to achieve optimal algorithm performance; lower values yield superior fitness results.

### Ablation study 5: stability of proposed model

The stability of the proposed scheme is validated through statistical analysis of the stored results based on fitness values, accuracy, and computational time obtained by the Monte Carlo simulations performed for 200 independent executions of the proposed model. By applying the relations given in relation (14) to (17) for $\overline{\mu }$, $\sigma$, $\widehat{\kappa }$ And $\Xi$, respectively, the results are tabulated in Table 9. The table statistically summarizes fitness, accuracy, and computational time to illustrate their performance and variability. The fitness parameter values span from a minimum of 1.4219 × 10^–9^ to a maximum of 1.0047 × 10^–7^, with a mean of 4.9930 × 10^–8^ and a standard deviation of 2.8657. The κ of 1.8327 signifies a positively skewed distribution, whereas the kappa coefficient (Ξ = 0.80) denotes robust measurement reliability.

**Table 9:** Global performance and stability of the proposed model.

Statistical parameters	Fitness	Accuracy (%)	Computational time
Min	1.4219e-09	**99.47**	600
Max	1.0047e-07	**99.55**	750
$\overline{\mu }$	4.9930e-08	**99.5**	678.910
$\sigma$	2.8657e-08	**0.067**	42.574
$\widehat{\kappa }$	1.8327	**1.5**	1.8508
$\Xi$	**0.80**	**0.81**	0.79

Significant values are in bold.

The model has high accuracy, varying from 99.47% to 99.55%, with an average accuracy of 99.5% and a minimal σ = 0.067, signifying reliable classification results. The computation duration varies from 600 to 750 s, with a mean of 678.910 s and a standard deviation of 42.574 s, signifying considerable variability. The skewness (κ = 1.8508) indicates a slight inclination towards shorter computational durations; however, the consistency (Ξ = 0.79) demonstrates the model’s efficacy. The table presents a very precise and computationally efficient system that yields consistent outputs across multiple runs.

### Comparison with state-of the-art and discussion

Table 10 delineates the accuracy, FNR and MET in seconds for proposed as well state-of-the-art reported results for the classification of the EuroSAT dataset through segmentation. ViT-SE hybrid with MFA achieves optimal performance in accuracy and error reduction, with an accuracy of 99.5%, a false negative rate of 0.5%, and a processing duration of 678.91 s. Despite achieving a quicker time of 651.279 s, the Fuzzy DL + chaotic PSO method^23^ exhibits a reduced accuracy of 93.3% and an elevated false negative rate of 6.7%. Deep CNN^22^ achieves an accuracy of 86%, accompanied by a 14% false negative rate and a mean execution time of 1273.651 s, indicating inefficiencies in both accuracy and computational performance. The Geosystem Approach exhibits a moderate accuracy of 91.52% and a false negative rate of 8.48%. Likewise, Gaussian Naïve Bayes^20^ attains an accuracy of 88.89% and a false negative rate of 11.11%. According to accuracy and balance, ViT-SE + MFA is the most effective approach for the task.

**Table 10:** Comparison of the proposed architecture with state-of-the-art and reported techniques.

Method applied	References	Performance measure		
		Accuracy (%)	FNR (%)	MET (sec)
ViT-SE + MFA	**Proposed**	**99.5**	**0.5**	**678.910**
Fuzzy DL + chaotic PSO	[23]	93.30	6.70	651.279
Deep CNN	[22]	86.00	14.0	1273.651
Geosystem approach	[21]	91.52	8.48	–
Gaussian naïve bayes	[20]	88.89	11.11	–

Significant values are in bold.

**Fig. 10:**
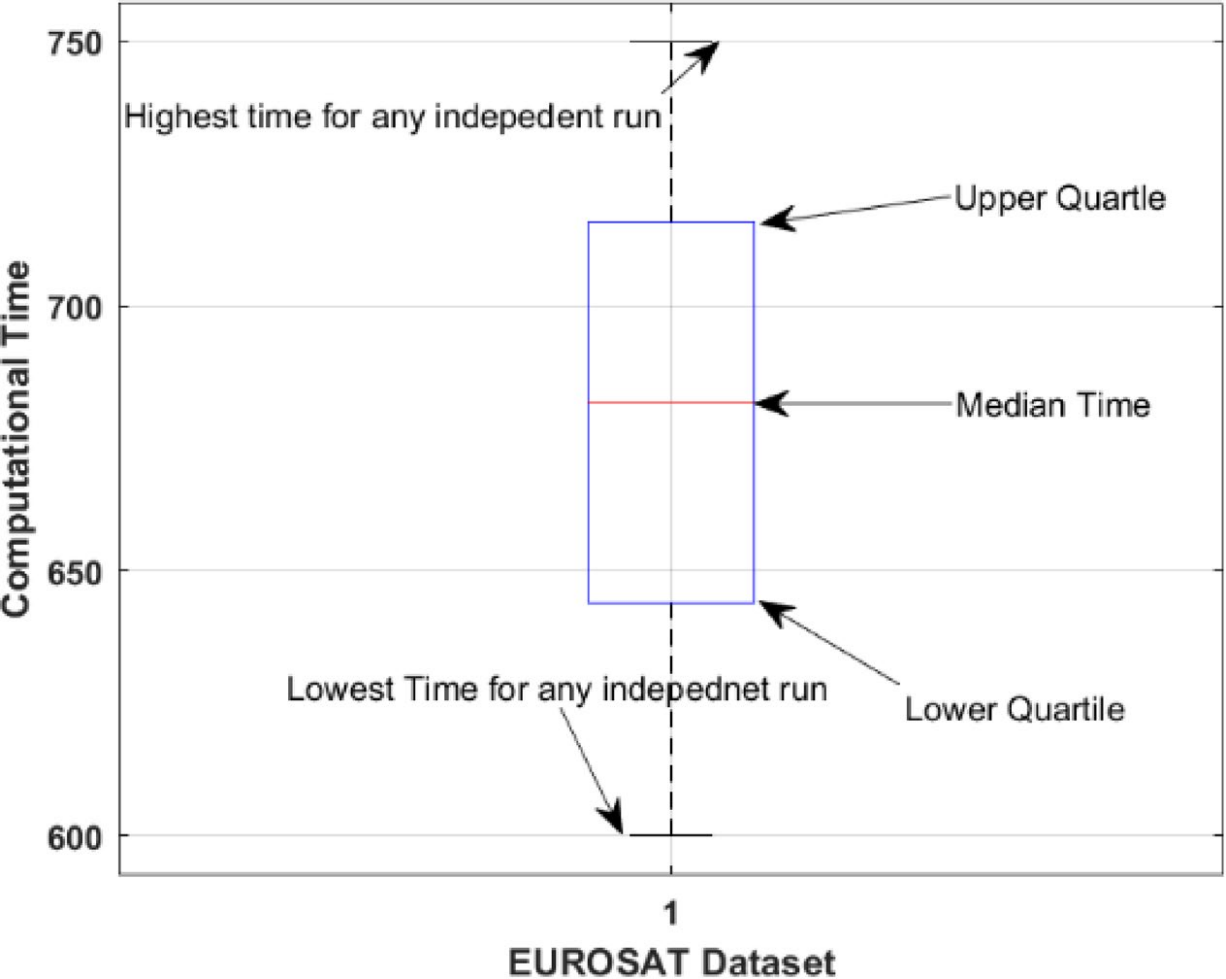
Box whisker analysis of computational time.

Along with the discussion on comparison with the state-of-the-art techniques, the behavior of computational time, as given in Fig. 8 and the impact of the proposed model on the learnable parameters, as tabulated in Table 11, are also presented. The box plot in Fig. 10 illustrates the distribution of computational time (in seconds) for independent runs of the EUROSAT dataset. The interquartile range encompassing the central 50% of computing times from the 25th to the 75th percentile indicates the algorithm’s runtime stability across the majority of executions. The whiskers extend from around 600 s to 750 s throughout all iterations, signifying a comprehensive data distribution devoid of outliers. The interquartile range is small within the central 50% of the data, indicating that most runs exhibit consistent computational time. The whiskers exhibit occasional fluctuations, as certain runs may be significantly longer or shorter. This figure demonstrates the algorithm’s computational efficiency and stability in processing the EUROSAT dataset.

**Table 11:** Comparison of the proposed model with pre-trained and reported result.

Technique	Learnable parameters (Millions)	Depth	Size (Mb)
Proposed	**4.3**	**49**	**2.9**
Fuzzy DL + chaotic PSO	5.7	67	34
VGG-19,	144	19	535
DarkNet-19	20.8	19	78
ResNet-50,	25.6	50	96
Inception-V3	23.9	48	89

Significant values are in bold.

Table 11 contrasts methodologies based on learnable parameters (in millions), depth (in layers), and size (in megabytes). The proposed method is the most lightweight among the mentioned techniques, comprising merely 4.3 million parameters, 49 layers, and 2.9 MB, hence ensuring computational and storage efficiency. The Fuzzy DL combined with chaotic PSO has 5.7 million parameters, 67 layers, and occupies 34 MB, indicating increased complexity and resource requirements. Conventional architectures such as VGG-19 are resource-demanding, comprising 144 million parameters, 19 layers, and occupying 535 MB of storage. DarkNet-19 and ResNet-50, with 20.8 million and 25.6 million parameters and sizes of 78 MB and 96 MB, respectively, provide greater depth. Inception-V3 features 23.9 million parameters, comprises 48 layers, and has a size of 89 MB, effectively balancing depth and size. In comparison to more advanced architectures, the proposed technique is optimal for resource-limited applications because of its minimal parameter count and compact size.

The evaluation methodology of the proposed ViT-SE + MFA framework follows a rigorous protocol to ensure robust and reproducible results. The dataset was split into 80% training and 20% testing, maintaining class balance across the EuroSAT categories. Evaluation was performed using accuracy, precision, recall, F1-score, mIoU, and Dice coefficient, alongside kappa statistics to assess agreement beyond chance. Additionally, Monte Carlo simulations with 200 independent runs were conducted to evaluate stability, and statistical significance of performance differences with respect to competing methods was verified using paired t-tests at a 95% confidence level (*p* < 0.05). Comparative analysis (Tables 10, 12) demonstrates that the proposed model improves classification accuracy by up to 6.2% and mIoU scores by up to 13.4% over the best-performing baseline, while reducing learnable parameters by more than 98% compared to traditional CNNs (e.g., VGG-19). These gains are attributed to the integration of SE modules within the ViT architecture for enhanced channel recalibration and the MFA optimizer for hyperparameter tuning, which collectively yield a lighter, faster, and more accurate model. This combination has not bee.

**Table 12:** Comparison with various segmentation models based on Miou.

Nature of the field	Methods			
	ENet^81^	DDRNet23^82^	U-Net^78^	Proposed
Agriculture	0.800	0.845	0.950	**0.984**
Forest	0.754	0.778	0.890	**0.957**
Water	0.731	0.772	0.810	**0.915**
Barren	0.494	0.585	0.750	**0.892**

Significant values are in bold.

Moreover, the mIoU segmentation models for agriculture, forest, water, and barren terrain in rural areas are also examined and compared with state-of-the-art existed techniques like ENet^81^, DDRNet23^82^ and U-Net^78^. The proposed model outperforms U-Net (0.950), ENet (0.800), and DDRNet23 (0.845) in agriculture with a mIoU of 0.984. For forest segmentation, it improves by 6.7% over U-Net (0.890) to 0.957. The proposed architecture outperforms U-Net (0.810) in water body segmentation with 0.915, a 10.5% improvement and similarly show the dominancy for author land covers. Therefore, it is quite evident from Table 12 that the proposed framework outperforms state-of-the-art methods and is ideal for rural land segmentation and environmental analysis, resulting in more accurate and reliable land-use classification.

In order to validate the strength of the proposed model, a comparison with lightweight segmentation models like Bilateral Segmentation Network (BiSeNet V2) that combines a spatial path for high-resolution detail and a context path for semantic information and SegFormer that uses mix ViT backbone with lightweight decoder is carried out. The comparative results of the BiSeNet V2, SegFormer and proposed architecture are tabulated in Table 13 to further.

**Table 13:** Comparison of the proposed model with lightweight segmentation models.

Nature of the field	Methods		
	SegFormer^83^	BiSeNet V2^84^	Proposed
Agriculture	0.9662	0.9298	**0.984**
Forest	0.9439	0.9228	**0.957**
Water	0.8903	0.8749	**0.915**
Barren	0.9042	0.8737	**0.892**

Significant values are in bold.

Highlight the advantages of the proposed method in resource-constrained scenarios. It is worth mentioning that the model size of SegFormer is observed to be larger than that of BiSeNet V2. However, the accuracy of BiSeNet V2 is higher than the SegFormer and lesser than the proposed architecture. Moreover, the comparison is also made by the proposed architecture and existed state of art DL segmentation models like Mask2Former^72^, One Former^73^, Swin-UNet^74^ and MP Former^75^. Table 14 presents a comparative evaluation of the proposed segmentation model against several state-of-the-art DL segmentation methods using three standard performance indicators: mean pixel accuracy (mPA), mean Intersection over Union (mIoU), and Panoptic Quality (PQ). The results show that the proposed model achieves the highest performance across all metrics, with an mPA of 0.9732, mIoU of 0.9834, and PQ of 0.9773, indicating superior pixel-level classification, region overlap accuracy, and overall segmentation quality. In comparison, Mask2Former demonstrates relatively lower performance (mPA = 0.8347, mIoU = 0.8376, PQ = 0.8364), while OneFormer and Swin-UNet show competitive but still lower results than the proposed model. Among the benchmarks, MP-Former performs closely, with mIoU and PQ values above 0.96, yet the proposed model still outperforms it marginally. Overall, these results confirm the robustness and effectiveness of the proposed approach in delivering more accurate and consistent segmentation compared to recent deep learning methods.

**Table 14:** Comparison of the proposed model with DL segemenattion models.

Methods	Segementation performance indicators		
	mPA	mIoU	PQ
Proposed	**0.9732**	**0.9834**	**0.9773**
Mask2Former^72^	0.8347	0.8376	0.8364
One Former^73^	0.9565	0.9435	0.9563
Swin-UNet^74^	0.9218	0.9282	0.9193
MP Former^75^	0.9635	0.9635	0.9654

Significant values are in bold.

## Conclusions

Keeping in view the simulations performed and their results in the form of the figures and table, the following conclusions are drawn:

- The uniqueness of the proposed Vit-SE mimic with the MFA approach is in terms of learnable parameters, the accuracy achieved, and the mIOU obtained in a reasonable computational budget. The framework exploits only 4.3 million learnable parameters and 2.9 megabytes of the size on the memory during the applicability that is quite compatible than that of pre-trained and reported results.
- The proposed method presents classification accuracy of 99.5% with an FNR of 0.5%, which is better than that of the reported results of Gaussian Naïve Bayes, geosystem approach, deep CNN, fuzzy DL hybrid with chaotic PSO having an accuracy of 89.96%, 91.52%, 86% and 93.3%, respectively.
- The model exhibits optimal performance at ξ = 0.0001 by achieving 99.50% accuracy, 0.9919 precision, 0.9912 recall, and 0.9953 F1-score, respectively. The mean fitness evaluation of MFA was concluded to be 4.9930 × 10^–8^ in MET of 678.910 s.
- The Monte Carlo simulations and the consequent tables and figures that are drawn, ensure the stability and reliability of the proposed architecture for the successful classification of EuroSAT data that is important for rural architecture development and planning within a reasonable computational time. In future, one can look into the hardware implementation of the proposed framework on nano chips.
- Deep learning-based segmentation and classification on EuroSAT is sensitive to spatial resolution and domain change. EuroSAT is obtained on Sentinel-2 satellite imagery. Therefore lighting, seasonal changes, atmospheric disturbances, and geographical variances can affect model performance in new sites.

In the future, real-world feature extraction or segmentation of pure pixels (annual crop, highway, river, forest) and one mixed-pixel region will also be considered to have an applied nature of the work.

## Data Availability

The dataset used in the study is accessible from the following link: https://www.kaggle.com/datasets/apollo2506/eurosat-dataset.
